# ISCEV extended protocol for the photopic negative response (PhNR) of the full-field electroretinogram

**DOI:** 10.1007/s10633-018-9638-x

**Published:** 2018-05-31

**Authors:** Laura Frishman, Maja Sustar, Jan Kremers, J. Jason McAnany, Marc Sarossy, Radouil Tzekov, Suresh Viswanathan

**Affiliations:** 1 College of Optometry, University of Houston, Houston, TX USA; 2 Eye Hospital, University Medical Centre Ljubljana, Ljubljana, Slovenia; 3 Department of Ophthalmology, University Hospital Erlangen, Erlangen, Germany; 4 Department of Ophthalmology and Visual Sciences, Department of Bioengineering, University of Illinois at Chicago, Chicago, IL USA; 5 Department of Ophthalmology, Centre for Eye Research Australia, The Royal Victorian Eye and Ear Hospital, University of Melbourne, Melbourne, VIC Australia; 6 Department of Ophthalmology, University of South Florida, Tampa, FL USA; 7 College of Optometry, State University of New York, New York, NY USA

**Keywords:** Clinical standards, Electroretinogram (ERG), Full-field ERG, International Society of Clinical Electrophysiology of Vision (ISCEV), Photopic negative response, PhNR, Optic neuropathy, Glaucoma, Retinal ganglion cells

## Abstract

The International Society for Clinical Electrophysiology of Vision (ISCEV) Standard for full-field electroretinography (ERG) describes a minimum procedure, but encourages more extensive testing. This ISCEV extended protocol describes an extension to the ERG Standard, namely the photopic negative response (PhNR) of the light-adapted flash ERG, as a well-established technique that is broadly accepted by experts in the field. The PhNR is a slow negative-going wave after the *b*-wave that provides information about the function of retinal ganglion cells and their axons. The PhNR can be reduced in disorders that affect the innermost retina, including glaucoma and other forms of optic neuropathy. This document, based on existing literature, provides a protocol for recording and analyzing the PhNR in response to a brief flash. The protocol includes full-field stimulation, a frequency bandwidth of the recording in which the lower limit does not exceed 0.3 Hz, and a spectrally narrowband stimulus, specifically, a red flash on a rod saturating blue background. Suggested flash strengths cover a range up to and including the minimum required to elicit a maximum amplitude PhNR. This extended protocol for recording the PhNR provides a simple test of generalized retinal ganglion cell function that could be added to standard ERG testing.

## Introduction

The International Society for Clinical Electrophysiology of Vision (ISCEV) Standard for full-field electroretinography (ERG) describes a minimum set of tests, but encourages the use of additional ERG protocols for clinical ERG testing [1]. This extended protocol describes the photopic negative response (PhNR) of the flash ERG, as a specialized procedure which is well established and broadly accepted by experts in the field. The protocol was prepared by the authors in accordance with ISCEV procedures (http://www.iscev.org/standards/index.html.) and was approved by the ISCEV Board of Directors on March 25, 2018.

## Scope and applications

The photopic negative response (PhNR) of the light-adapted (LA) electroretinogram (ERG) is a negative-going wave that occurs after the *b*-wave in response to a brief flash. The PhNR reflects generalized activity of retinal ganglion cells and their axons [2], and its amplitude can be reduced early in diseases that affect the innermost retina. The PhNR also occurs in response to long-duration flashes, following the *b*-wave at light onset and *d*-wave at light offset [3], but most publications to date have described brief flashes. Only the brief flash PhNR will be addressed in this protocol.

## Patient population

**Fig. 1:**
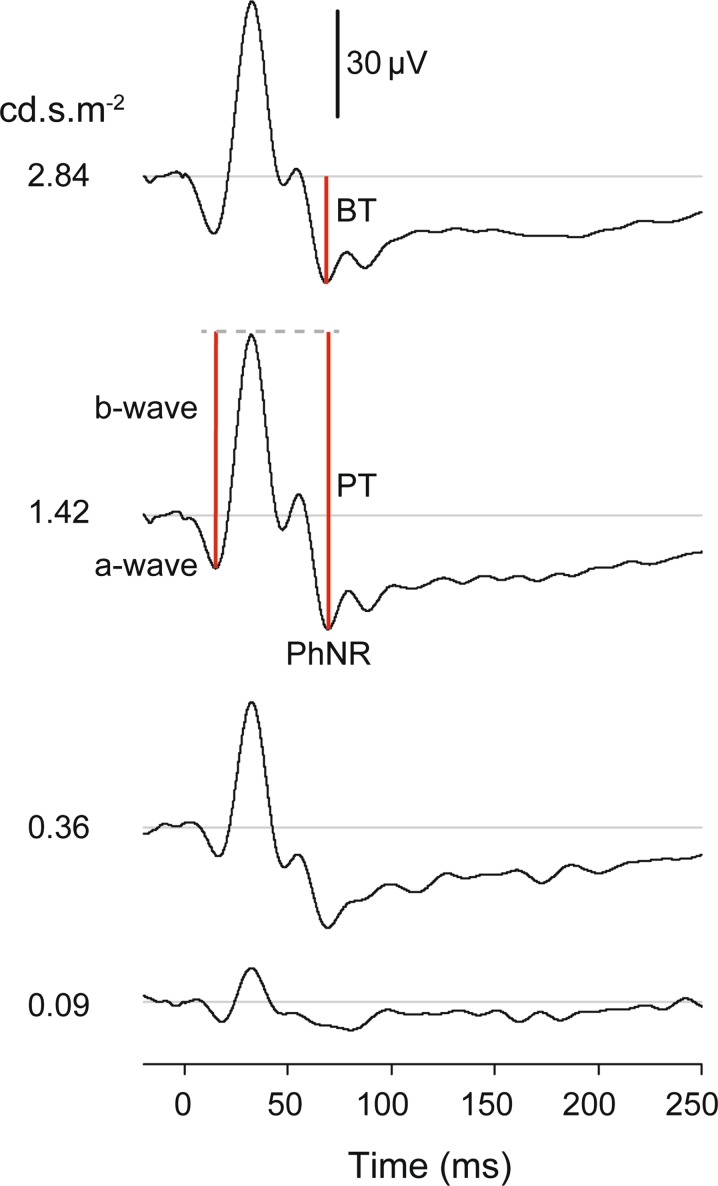
Illustration of the light-adapted ERG of a healthy subject (35 years.) in response to a brief red LED flash (660 nm) at each of four flash strengths, on a blue background (460 nm) of 10 cd m^−2^. Figure shows PhNR amplitude measurements from baseline to PhNR trough (BT) and from *b*-wave peak to PhNR trough (PT). Adapted from Ref. [26] (the Association for Research in Vision and Ophthalmology is the copyright holder)

This protocol for recording the PhNR can be used for testing patients in whom inner retinal integrity, and specifically signaling by retinal ganglion cells and their axons, may be compromised due to ganglion cell pathology or limitations in the input to the ganglion cells. For example, since 2000, reduced PhNR amplitudes have been reported in patients with glaucoma [3–6], optic atrophy [7, 8], central retinal artery occlusion [9, 10], ischemic optic neuropathy [11], diabetic retinopathy [12], and idiopathic intracranial hypertension [13]. In some cases, the protocol may be useful for monitoring treatment effects in eyes with ocular hypertension or glaucoma [14]. Abnormal potassium (K^+^) channel activity or other dysfunction of retinal glia may also be reflected in PhNR recordings [15]. This is because generation of the PhNR, which has a slow time course (Fig. 1), is thought to involve glial K^+^ currents that serve to remove the excess K^+^ released into extracellular space during activation of retinal ganglion cells [16].

## Technical issues

The electrodes and electronic recording equipment for this PhNR protocol are as described in the ISCEV Standard for full-field ERG [1]. The present protocol assumes full-field stimulation, while acknowledging that focal stimulation has been shown to be effective in assessing inner retinal function [17]. For the frequency bandwidth of the recording, the ISCEV Standard suggests a minimum range of 0.3–300 Hz. For PhNR recordings, the bottom limit of the filtering could be lower to minimize distortion and possible attenuation of the slow negative wave. For spectral characteristics of the stimulus, whereas the ISCEV Standard recommends “visibly white” (broadband) stimuli, narrowband stimuli are recommended for recording the PhNR. Specifically, a long-wavelength (red) flash on a rod saturating short-wavelength (blue) background yields a larger amplitude PhNR than broadband stimuli. LED-based stimulators typically provide a 20-nm half-height bandwidth for the red and blue LEDs. The recommendation for narrowband stimuli is based on the outcome of studies that compared PhNR amplitudes using broad- vs narrowband stimuli in nonhuman primates [18] and in glaucoma patients [6, 18, 19], and more generally on a review of the literature which shows that most studies in patients have used red LED flashes on blue LED backgrounds. It should be noted that other narrowband combinations using blue flashes on yellow or orange backgrounds have also been reported to be effective for eliciting a robust PhNR [19, 20].

## Calibration

The stimulus strength for the brief flashes can be specified in photopic candela seconds per meter squared (phot cd s m^−2^); the background in phot cd m^−2^. A spectroradiometer (or spectrometer) is required to determine the spectral characteristics of chromatic flashes. Care should be taken to measure a range of flash luminances as some Ganzfeld stimulators use different combinations and banks of LEDs for different luminance ranges, and these may have different wavelength specifications. It is useful also to confirm that the background is strong enough to saturate rod photoreceptors, for example, about 100 scot cd m^−2^. Blue backgrounds will saturate the rods while minimizing the photopic stimulus strength and hence the adapting effect of the background on cone-driven responses.

## Protocol specifications

The procedures for patient preparation and recording are as specified by the ISCEV Standard for the light-adapted ERG, including pupil dilation and 10 min of light adaptation if the patient was dark adapted for other testing prior to recording the light-adapted ERG. Other specifications are listed below;

- The chromatic characteristics of the stimuli. Background: steady, blue LED (450–485 nm); 100 scot cd m^−2^; equivalent to ~ 10 phot cd m^−2^. Light flash: red LED (630–660 nm).
- Flash strengths and background luminance. Flash: < 5 ms; 1.0–2.5 phot cd s m^−2^, or the stimulus strength that produces the largest PhNR amplitude, but does not exceed the initial stimulus strength producing amplitude saturation, or lead to the decline in response amplitude associated with the photopic hill [21, 22]. The dynamic range of the stimulus response function generally ranges from ~ 0.01 to > 2.0 phot cd s m^−2^.
- Frequency of flash presentation. Inter-flash interval: 1 s. Some studies have used an interval of 500 ms, but this may not allow enough time for PhNR to fully recover to baseline.
- Recording bandwidth. The low-frequency filter should be 0.3 Hz or lower; the high-frequency filter, a minimum of 300 Hz.
- Signal averaging. There should be sufficient repetitions to provide good signal-to-noise ratio, and many studies have used 20 trials or more. At least 8–10 trials or more are necessary for lower stimulus strengths if a range of stimuli are used that include weak stimuli, fewer may be necessary for saturated responses. Artifact rejection should be used if available. If single responses are saved, noisy responses can be removed during off-line analysis before averaging.

## Response evaluation

As shown in Fig. 1, the PhNR amplitude can be measured from baseline to the minimum point in the trough (BT). It also can be measured from the peak of the *b*-wave to the maximum amplitude in trough (PT). Alternatively, PhNR amplitude can be measured at a fixed time, for example, at 65–75 ms after the flash in the trough of the response (not shown). Using a fixed time could be helpful when responses in diseased eyes are small and the trough is difficult to locate. Note that the PT measurement is largely dominated by the *b*-wave amplitude, and a change in *b*-wave amplitude reflecting a change in bipolar cell function must be considered when interpreting a change in PhNR amplitude. When measuring the PhNR, it may also be necessary to take account of the *i*-wave, or *i*-waves, positive deflection(s) of Off pathway origin [11] in the falling limb of the *b*-wave, and/or later in the trough (Fig. 1). For responses to the suggested narrowband stimuli, such as those used for responses in Fig. 1, the maximum trough amplitude generally occurs after the initial *i*-wave. Given the slow nature of the response, and the variety of amplitude criteria that have been used, peak time of the PhNR is generally not reported. The PhNR is moderately affected by age, so, for the particular measure(s) chosen, appropriate age-matched normative data should be used [3, 22]. Comparisons of longitudinal findings in patients to normal test-retest repeatability of PhNR amplitudes are also important, as the test–retest variability of PhNR amplitudes can be greater than that of *a*- and *b*-waves [21–24].

## Reporting

Reporting of results of PhNR testing should include measurements of the *a*-wave, *b*-wave, and PhNR and a computation of the PhNR: *b*-wave ratio. This helps to determine whether the origin of any change in PhNR amplitude is at the retinal ganglion cells themselves or a more distal location in the retina. The choice of method for measuring PhNR amplitude is open to the study and the site, but for comparison with other studies, inclusion of the BT measure is advised. Some studies have compared the sensitivity of the ratio of PhNR to *b*-wave amplitude (i.e., PhNR normalized to *b*-wave) versus the simple BT measure for detecting glaucoma, and results were mixed [5, 25]. Caution is needed as the ratio measure could be misleading in diseases where the *b*-wave is abnormal.

## Appendix: Justification for the protocol details

A systematic literature review was performed using PubMed to find publications that reported use of the PhNR from the period 1999–2017. The committee identified the relevant references to include in the reference list, discussed the methods used in the references to record PhNRs, and came to a consensus on those to include in the extended protocol.

## References

[1] McCulloch DL, Marmor MF, Brigell MG, Hamilton R, Holder GE (2015). ISCEV Standard for full-field clinical electroretinography (2015 update). Doc Ophthalmol.

[2] Viswanathan S, Frishman LJ, Robson JG, Harwerth RS, Smith EL (1999). The photopic negative response of the macaque electroretinogram: reduction by experimental glaucoma. Invest Ophthalmol Vis Sci.

[3] Viswanathan S, Frishman LJ, Robson JG, Walters JW (2001). The photopic negative response of the flash electroretinogram in primary open angle glaucoma. Invest Ophthalmol Vis Sci.

[4] Colotto A, Falsini B, Salgarello T, Iarossi G, Galan ME (2000). Photopic negative response of the human ERG: losses associated with glaucomatous damage. Invest Ophthalmol Vis Sci.

[5] Machida S, Gotoh Y, Toba Y, Ohtaki A, Kaneko M (2008). Correlation between photopic negative response and retinal nerve fiber layer thickness and optic disc topography in glaucomatous eyes. Invest Ophthalmol Vis Sci.

[6] Sustar M, Cvenkel B, Brecelj J (2009). The effect of broadband and monochromatic stimuli on the photopic negative response of the electroretinogram in normal subjects and in open-angle glaucoma patients. Doc Ophthalmol.

[7] Gotoh Y, Machida S, Tazawa Y (2004). Selective loss of the photopic negative response in patients with optic nerve atrophy. Arch Ophthalmol.

[8] Miyata K, Nakamura M, Kondo M, Lin J, Ueno S (2007). Reduction of oscillatory potentials and photopic negative response in patients with autosomal dominant optic atrophy with OPA1 mutations. Invest Ophthalmol Vis Sci.

[9] Machida S, Gotoh Y, Tanaka M, Tazawa Y (2004). Predominant loss of the photopic negative response in central retinal artery occlusion. Am J Ophthalmol.

[10] Shinoda K, Yamada K, Matsumoto CS, Kimoto K, Nakatsuka K (2008). Changes in retinal thickness are correlated with alterations of electroretinogram in eyes with central retinal artery occlusion. Graefes Arch Clin Exp Ophthalmol.

[11] Rangaswamy NV, Frishman LJ, Dorotheo EU, Schiffman JS, Bahrani HM (2004). Photopic ERGs in patients with optic neuropathies: comparison with primate ERGs after pharmacologic blockade of inner retina. Invest Ophthalmol Vis Sci.

[12] Chen H, Zhang M, Huang S, Wu D (2008). The photopic negative response of flash ERG in nonproliferative diabetic retinopathy. Doc Ophthalmol.

[13] Moss HE, Park JC, McAnany JJ (2015). The photopic negative response in idiopathic intracranial hypertension. Invest Ophthalmol Vis Sci.

[14] Niyadurupola N, Luu CD, Nguyen DQ, Geddes K, Tan GX (2013). Intraocular pressure lowering is associated with an increase in the photopic negative response (PhNR) amplitude in glaucoma and ocular hypertensive eyes. Invest Ophthalmol Vis Sci.

[15] Thompson DA, Feather S, Stanescu HC, Freudenthal B, Zdebik AA (2011). Altered electroretinograms in patients with KCNJ10 mutations and EAST syndrome. J Physiol.

[16] Raz-Prag D, Grimes WN, Fariss RN, Vijayasarathy C, Campos MM (2010). Probing potassium channel function in vivo by intracellular delivery of antibodies in a rat model of retinal neurodegeneration. Proc Natl Acad Sci U S A.

[17] Machida S, Toba Y, Ohtaki A, Gotoh Y, Kaneko M (2008). Photopic negative response of focal electoretinograms in glaucomatous eyes. Invest Ophthalmol Vis Sci.

[18] Rangaswamy NV, Shirato S, Kaneko M, Digby BI, Robson JG (2007). Effects of spectral characteristics of ganzfeld stimuli on the photopic negative response (PhNR) of the ERG. Invest Ophthalmol Vis Sci.

[19] Kremers J, Jertila M, Link B, Pangeni G, Horn FK (2012). Spectral characteristics of the PhNR in the full-field flash electroretinogram of normals and glaucoma patients. Doc Ophthalmol.

[20] Drasdo N, Aldebasi YH, Chiti Z, Mortlock KE, Morgan JE (2001). The s-cone PHNR and pattern ERG in primary open angle glaucoma. Invest Ophthalmol Vis Sci.

[21] Binns AM, Mortlock KE, North RV (2011). The relationship between stimulus intensity and response amplitude for the photopic negative response of the flash electroretinogram. Doc Ophthalmol.

[22] Joshi NR, Ly E, Viswanathan S (2017). Intensity response function of the photopic negative response (PhNR): effect of age and test-retest reliability. Doc Ophthalmol.

[23] Mortlock KE, Binns AM, Aldebasi YH, North RV (2010). Inter-subject, inter-ocular and inter-session repeatability of the photopic negative response of the electroretinogram recorded using DTL and skin electrodes. Doc Ophthalmol.

[24] Tang J, Edwards T, Crowston JG, Sarossy M (2014). The test–retest reliability of the photopic negative response (PhNR). Transl Vis Sci Technol.

[25] Preiser D, Lagreze WA, Bach M, Poloschek CM (2013). Photopic negative response versus pattern electroretinogram in early glaucoma. Invest Ophthalmol Vis Sci.

[26] Wang J, Cheng H, Hu YS, Tang RA, Frishman LJ (2012). The photopic negative response of the flash electroretinogram in multiple sclerosis. Invest Ophthalmol Vis Sci.

